# Fluorescent Aptamer Immobilization on Inverse Colloidal Crystals

**DOI:** 10.3390/s18124326

**Published:** 2018-12-07

**Authors:** Andrea Chiappini, Laura Pasquardini, Somayeh Nodehi, Cristina Armellini, Nicola Bazzanella, Lorenzo Lunelli, Stefano Pelli, Maurizio Ferrari, Silvia M. Pietralunga

**Affiliations:** 1 CNR-IFN CSMFO Lab. and FBK CMM, via alla Cascata 56/C, 38123 Povo Trento, Italy; andrea.chiappini@unitn.it (A.C.); cristina.armellini@unitn.it (C.A.); maurizio.ferrari@unitn.it (M.F.); 2 FBK-LaBSSAH, via Sommarive 18, 38123 Povo Trento, Italy; lunelli@fbk.eu; 3 Indivenire Srl, via Alla Cascata 56/C, 38123 Povo Trento, Italy; 4 CNR-IFN Milano, piazza Leonardo da Vinci 32, 20133 Milano, Italy; somayehnodehi@gmail.com (S.N.); silvia.pietralunga@polimi.it (S.M.P.); 5 Physics Department, University of Trento, via Sommarive 14, 38123 Trento, Italy; nicola.bazzanella@unitn.it; 6 Institute of Biophysics, CNR, via Sommarive 18, 38123 Povo Trento, Italy; 7 IFAC—CNR, MiPLab., via Madonna del Piano10, 50019 Sesto Fiorentino, Italy; s.pelli@ifac.cnr.it; 8 Enrico Fermi Centre, piazza del Viminale 1, 00184 Roma, Italy

**Keywords:** colloidal crystal, fluorescence, band gap, co-assembly, DNA-aptamers, FDTD simulations, PWE method

## Abstract

In this paper, we described a versatile two steps approach for the realization of silica inverse opals functionalized with DNA-aptamers labelled with Cy3 fluorophore. The co-assembly method was successfully employed for the realization of high quality inverse silica opal, whilst the inverse network was functionalized via epoxy chemistry. Morphological and optical assessment revealed the presence of large ordered domains with a transmission band gap depth of 32%, after the functionalization procedure. Finite Difference Time-Domain (FDTD) simulations confirmed the high optical quality of the inverse opal realized. Photoluminescence measurements evidenced the effective immobilization of DNA-aptamer molecules labelled with Cy3 throughout the entire sample thickness. This assumption was verified by the inhibition of the fluorescence of Cy3 fluorophore tailoring the position of the photonic band gap of the inverse opal. The modification of the fluorescence could be justified by a variation in the density of states (DOS) calculated by the Plane Wave Expansion (PWE) method. Finally, the development of the aforementioned approach could be seen as proof of the concept experiment, suggesting that this type of system may act as a suitable platform for the realization of fluorescence-based bio-sensors.

## 1. Introduction

Photonics-based biosensor platforms have demonstrated additional performance qualities compared to other sensing methods that rely on electrical, mechanical, or mass spectroscopic techniques. The main advantages of the photonics-based biosensor platforms can be attributed to the platforms response time, the immunity of the signal to electrical and magnetic interference, and the potential for higher information content. A large variety of optical methods have been used in biosensors. Devices based on surface plasmon resonance [1], interferometry and spectroscopy of guided modes in optical waveguide structures (grating coupler and resonant mirror) [2], fluorescence resonant energy transfer and fluorescence amplification by photonic crystals [3,4], whispering gallery modes in functionalized microresonators [5], and microfluidic devices [6], have been developed.

Among the different types of optical systems, Photonic Crystals (PCs) have attracted considerable attention due to the PCs optical features [7,8]. PCs are extremely dispersive structures, where spectral regions of forbidden optical propagation (Photonic Band Gaps-PBG) occur, due to the spatial periodicity of the dielectric constant over distances comparable to the operating optical wavelengths. Optical bandgaps can open either along the selected directions of light propagation (pseudo-PBG), or they can be omni-directional and completely prevent optical propagation (full-PBG). PBGs correspond to no-photon regions in the photon density of states (DOS) at given k-vectors, while strong modulations in the DOS occur at frequencies near to the forbidden spectrum [9], where the intensity of the optical field may be strongly enhanced. One of the consequences of the tailoring in the DOS performed by PCs is the modification and even the inhibition of the process of optical spontaneous emission (SE) of fluorescent species at critical frequencies [10]. Controlling the SE and photon management by PCs is appealing for the development of chemical sensing and biosensing devices [11,12,13].

In particular, 3D photonic crystals structures have shown to be potentially useful in a wide range of fields, including photonics [14,15,16], tissue engineering [17,18], catalysis [19,20], and the realization of biosensors [21]. Although there are several different top-down approaches to produce 3D photonic crystals [22], the chemical bottom-up methods are more simple, cheaper, and allow the obtaining of direct periodic colloidal crystals called the “opal” template, which in turn, can direct the infiltration and deposition of functional molecules to yield nanoporous “inverse opal” structures. Inverse opals exhibit a high degree of interconnected porosity (approximately 74%), with extremely uniform size (average size normally in the range of 100–1000 nm) and periodic distributions of pores, achieved through colloidal monodispersity. Zhao et al., have proposed a fabrication protocol for the realization of large area crack free colloidal photonic crystals based on the vertical deposition approach [23]. Based on this method, Teng and Zhao’s groups [24,25,26] have developed a co-assembly approach that allows the realization of more scalable robust, higher optical quality, and crack-free domains in dielectric inverse opal films, with respect to the conventional one [27]. A similar approach has been extended by Aizenberg et al., as reported in References [28,29]. These features combined with the immobilization of the specific bioassay layers can be exploited for the realization of biosensor platforms in a dye-labelled fluorescence detection scheme.

In this paper, we reported on a two steps approach for the realization of silica inverse opals functionalized with a DNA-aptamer sequence labelled with Cy3 fluorophore (DNA-Cy3). Aptamers are single-stranded nucleic acid molecules and represent a promising alternative to the traditional antibody-based approaches used in biomedical diagnosis and other biotechnological applications. The aptamers represent a highly versatile class of biosensing molecules, since they can be targeted to a wide variety of analytes [30,31]. They are also characterized by a high reproducibility in target recognition, as well as being prone to chemical modifications and being highly stable even in non-physiological conditions [31]. In this study, we employed a DNA-aptamer that was able to recognize the tumor necrosis factor-alpha (TNFα) [32], a primary pro-inflammatory cytokine that plays a crucial role in enhancing the immune response associated with degenerative diseases.

Some works report on the aptamer employment as a heavy metals recognition agent in combination with PC structures exploiting the variation of the Bragg diffraction peak position, as a function of the analytes to be detected. Ye et al. [33], for instance, exploited the use of colloidal nanoparticles that were forced to form a close-packed colloidal crystal infiltrated with a specific hydrogel able to shrink upon the stimulus. Based on the change in the diffraction peak wavelength position, Lee et al. [34] have also demonstrated the possibility to fabricate an optical biosensor that takes advantage of the silica inverse opal structure useful for the capture of influenza viruses; however, no characterizations have been performed on the ability to modify the complete volume of the structure. Moreover, the small reflectance peak shift that was measured (around 1 nm), requires a high-resolution spectrometer and can be heavily affected by the intrinsic polydispersity in the dimensions of the hollow spheres.

A complementary approach, for the detection of heavy metals, has been proposed by Zhang et al. [35], by exploiting the properties of an ssDNA functionalized metallo-dielectric structure, which exploits the fluorescence quenching. In this case, a direct photonic crystal is obtained and subsequently modified with a sputtered gold film; therefore, no infiltration all through the structure is obtained.

Choi et al., reported that the fluorescence of the colloidal crystal is restored after target binding, because this event implicates the de-hybridization of a complementary quencher-modified sequence that is responsible for the fluorescence reduction [36]. A similar approach was proposed by Zhou et al. [37], where the aptameric sequence was hybridized with a biotinylated complementary sequence, and after target binding, the streptavidin-modified quantum dots were used to detect the remaining un-reacted sequences. Both these approaches can be used in our system, which takes advantage of the higher reaction volume and the optical properties of PCs, such as the capability to modify the photon density of states. A recent work by Xu et al. [38] reports on the employment of hydrogel inverse opal particles decorated by aptamers, for the detection of bacteria in blood, using a barcode strategy based on the characteristics of the reflection peaks.

However, in these works, there were no references to the ability to functionalize the entire sample thickness reported. Lee et al. [39] reported on confocal microscopy on fluorescein-labelled streptavidin on inverse opal hydrogel, highlighting the protein immobilization across the whole structure.

The degree of difficulty in performing the aptamer immobilization on the entire sample thickness is underestimated, and the ability to homogenously functionalize the matrix is one of the key factors for the realization of reliable sensors.

From this perspective, an exhaustive structural and optical characterization is fundamental to assess the features of the inverse colloidal crystal, and to verify the effect of the functionalization procedure on the properties of the inverse opals. Finite difference time-domain (FDTD) simulations were performed to validate the spectral response of the structure.

Finally, numerical calculations, based on plane wave expansion method [40], permitted this study to determine the density of states and discuss the experimentally observed modification of the fluorescence of DNA-Cy3, confirming that the immobilization of the labelled DNA-Cy3 sequence occurred on the entire thickness of the inverse structure.

## 2. Experimental

### 2.1. Materials

Sodium dodecyl sulphate, potassium persulphate, sodium hydroxide, tetraethyl orthosilicate (TEOS), (3-Glycidyloxypropyl)trimethoxysilane (GPTMS), toluene and toluene anhydrous (99.8%), ethanolamine, and powders for buffered solutions were purchased from Sigma-Aldrich (St. Louis, MO, USA), and were used without any further purification. Styrene monomer was purified by washing it with sodium hydroxide solution to remove the polymerization inhibitor. The DNA-aptamer sequence (5′-/5AmMC12/TG GTG GAT GGC GCA GTC GGC GAC AA/3Cy3Sp/-3′), with amino modification in the 5′ end and Cy3 fluorophore in the 3′ end was HPLC purified, and it was purchased from IDT Integrated DNA Technologies (Leuven, Belgium).

### 2.2. Synthesis of Polystyrene Nanospheres

Colloidal polystyrene (PS) nanoparticles, used as building blocks for the fabrication of inverse silica opals, were synthesized by emulsion polymerization as described in Reference [12]. The remover of the polymerization inhibitor sodium hydroxide, the polymerization initiator potassium persulfate, and the surfactant sodium dodecyl sulfate, were used as received, but only the precursor styrene monomer was washed with 10% NaOH solution and water to remove the polymerization inhibitor.

The polymerization was carried out in a three necks glass reactor equipped with a condenser. The temperature of 80 °C was measured by a thermocouple immersed in the solution and connected to a heating jacket with a Proportional-Integral-Derivative (PID) controller. The solution was stirred by means of a mechanical stirrer that was set at 300 rpm. After four hours, the polymerization was complete, and the cooled suspension was centrifuged and washed several times to remove the unreacted chemicals.

### 2.3. Preparation of the Prehydrolized Silica Sol

As already mentioned, the inverse silica opals were obtained by a modified co-assembly method, which involved the deposition of polymeric colloidal nanospheres from a suspension also containing a silica sol, with the aim of producing a silica matrix in the interstitial space of the polystyrene spheres that would be removed by calcination in a second step.

The prehydrolized SiO_2_ sol was obtained by mixing TEOS, the silica precursor, with water and ethanol. Briefly, 5 mL of water were mixed with 5 mL of ethanol and added to 1.25 mL of TEOS. The solution was maintained under vigorous stirring for 1 h.

### 2.4. Deposition of Colloidal Crystal Films and Calcination

**Figure 1:**
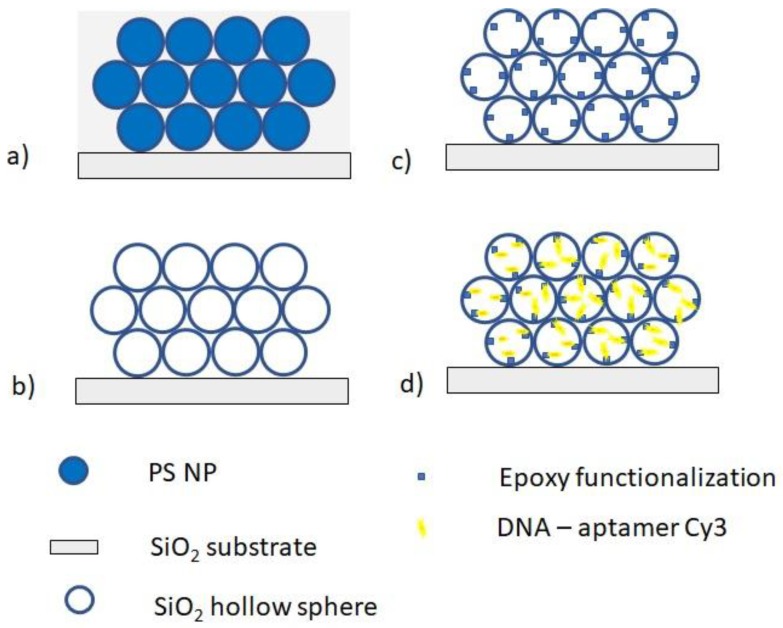
Sketch of the fabrication process for the realization of inverse silica opal and its functionalization with DNA-aptamer Cy3: (**a**) deposition of colloidal crystal film on the substrate, (**b**) calcinations of polystyrene template, (**c**) epoxy-functionalization of the colloidal film, (**d**) DNA-aptamer immobilization.

A suspension containing 150 µL of PS nanospheres, 5 mL of distilled water, and 80 µL of the abovementioned TEOS prehydrolized solution, was used to produce the colloidal film on vitreous silica substrates. The silica slide was vertically suspended in a vial containing the colloidal suspension. The slow evaporation of the solvents at 45 °C over a period of two days allowed the self-assembly of the colloidal particles at the meniscus and the consequent deposition of the colloidal crystal film on the substrate (see Figure 1a). Finally, an inverse silica opal was obtained by calcination of the polystyrene template. The film was firstly heated for 2 h at 200 °C with a heating rate of 0.5 °C/min, and then for 2 h at 450 °C with a rate of 2 °C/min (see Figure 1b).

### 2.5. Functionalization and Immobilization of the DNA-Cy3

The inverse silica opal surface was functionalized via epoxy-chemistry. After a cleaning procedure with argon plasma (6.8 W, one min), to remove organic contaminants and to hydroxylate the surface, the inverse silica opal (ISO) sample was treated with 0.1% (*v/v*) GPTMS in toluene anhydrous at 60 °C for 10 min, washed several times in toluene and then dried in a stream of nitrogen (see Figure 1c). Then, a 10 μM amino-modified DNA-aptamer solution was incubated (after heating at 95 °C for one minute) on the surface in a 0.05 M sodium phosphate buffer (ionic strength 300 mM, pH 8), for 3 h at room temperature (see Figure 1d). In this way, a covalently bound aptamer layer was deposited onto the functionalized PC surface. After a passivation step with 1 mM ethanolamine for 30 min, as well as extensive washing in buffer and a last one in ultrapure water, the inverse silica opal surface was left to dry in air.

### 2.6. Characterization

Optical properties of the inverse opals were evaluated by transmittance and reflectance measurements at different angles, using a double beam Varian spectrophotometer. Reflection spectra acquired at normal incidence were taken using a fiber-optic UV–VIS spectrometer (Ocean Optics, USB4000, Largo, FL, USA), with a beam spot of about 1 mm^2^ in area. SEM measurements were acquired using a JEOL JSM-7001F (JEOL, Tokyo, Japan), to determine the range of the ordered domains and the thickness of the inverse opal. A Leica SP5-II confocal microscope (Leica Instruments, Wetzlar, Germany), equipped with an He/Ne laser (543 nm) was used to verify the immobilization of the DNA-aptamer sequence. All the samples were observed using a 20X magnification objective, thereby obtaining images with an area of about 775 µm × 775 µm. The emission bandwidth was set from 547 nm up to 720 nm. Moreover, continuous wave (CW) luminescence measurements were performed by exciting the samples with the 514.5 nm line of an Ar^+^ laser, and the fluorescence from the sample was analyzed using a double monochromator and photon counting technique.

## 3. Results and Discussion

Inverse silica opals were realized using a modified co-assembly approach. This method with respect to the conventional one, which involves sequential steps (such as assembly of the colloids in an ordered structure, infiltration of the matrix, and finally removal of the beads), allowed the obtaining of larger ordered domains, reducing the cracks, but also yielding more robust inverse opals with no overlayer exploiting the gluing action of the sol-gel-derived matrix. These features are fundamental for the development of high optical quality inverse opal films, making them well suited for microfluidics, optical and photonic devices, or biosensors.

In this context, the realization of high performing sensors passes through an exhaustive morphological and optical characterization of the starting template defining the main features in terms of optical properties (position of the photonic band gap (PBG) as a function of the detection angle *θ*, frequency gap, and depth of the PBG) and morphological properties (dimension of the domains), where cracks and defects free structures allow the obtaining of a homogenous response over the entire system.

**Figure 2:**
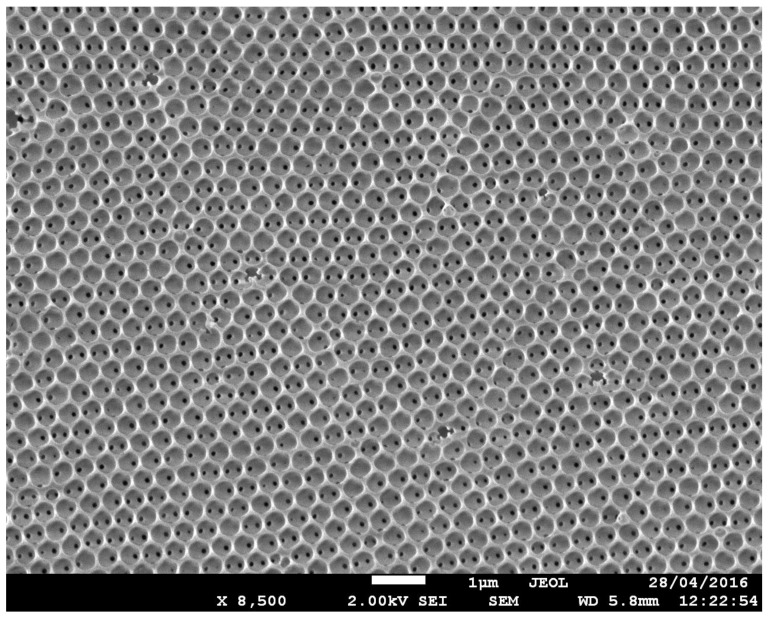
SEM image of inverse silica opal film obtained using co-assembly approach, showing high uniformity, with a diameter of the “holes” of about 370 nm.

Figure 2 shows the typical SEM image of the top surface of the inverse silica opal (ISO), where it is possible to observe a long order periodicity domain and a hexagonal arrangement attributable to the <111> plane of the fcc structure [41]. We could observe that the inverse ordered structure was constituted by hollow regions of air spheres and inner dark holes representing the point of contact between each templating sphere and its 12 nearest neighbors. The average size of the hollow spheres was about 370 nm. The cross section of the inverse opal is reported in Figure S2 in the ESI file.

Considering the structural parameters (such as the dimension of hollow spheres) revealed by the SEM image (Figure 2), numerical simulations were performed using a commercial FDTD Software package (Lumerical Solutions Inc., Vancouver, BC, Canada) to confirm the position of the passband in the reflection spectrum, obtained at an incident angle of 0° and 40°.

In our specific case, it was considered as an inverse structure constituted by air holes with a diameter D of 370 nm, embedded in a silica matrix, and assembled in a hexagonally close packed pattern, where the lattice constant *a* is:

(1) $$a=\sqrt{2}\cdot D$$

For this configuration, the effective refractive index is given by:

(2) $${\left(n_{eff}\right)}^{2}=f\cdot n_{air}^{2}+\left(1-f\right)\cdot n_{sn}^{2}$$

where *f* corresponds to the filling factor (0.74 in the present case) for a close-packed fcc lattice, and *c* is the speed of light. $n_{sn}^{2}=2.1$ is the silica dielectric function and $n_{air}^{2}$ = 1.

**Figure 3:**
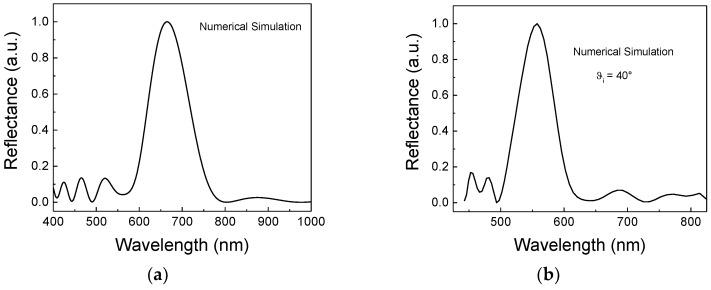

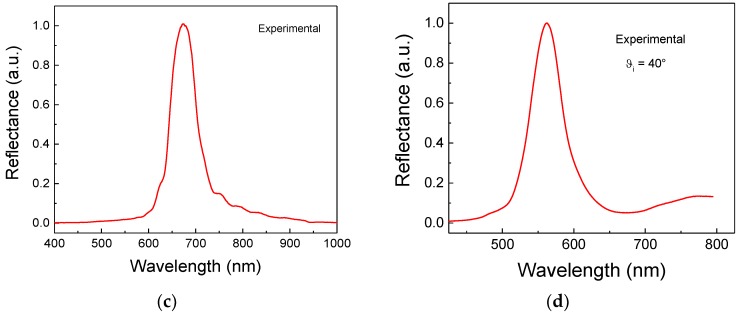
Simulated reflectance spectra (**a**) θ_i_ = 0° and (**b**) at θ_i_ = 40° obtained by the FDTD Software package. Experimental reflectance spectra at (**c**) θ_i_ = 0° and (**d**) at θ_i_ = 40°, respectively. θ_i_ is the incident angle.

Figure 3 summarizes the simulated reflectance spectra and the experimental ones.

A comparison between the graphs reported in Figure 3 revealed good agreement when considering the peak positions, which confirmed the excellent quality of the inverse opal that was realized.

**Figure 4:**
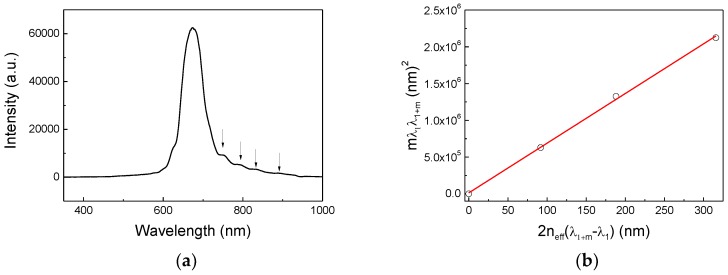
(**a**) Reflectance spectrum acquired at normal incidence on the inverse silica opal, the arrows correspond to the position of the Fabry-Perot fringes considered for the estimation of the thickness. (**b**) Experimental values of *mλ*_1_*λ*_1+*m*_ plotted as a function of 2*n_eff_*(*λ*_1+*m*_ − λ_1_) and the corresponding linear fit.

Moreover, the thickness of the inverse opal was determined by means of reflection measurements (Figure 4a). In fact, by analyzing the spectrum reported in Figure 4a it was possible to observe both the main peak attributed to the Bragg diffraction (*λ_b_*) and several peaks known as the Fabry-Perot (FP) oscillations for wavelengths higher than *λ_b_*, which were due to the interference of the light reflected by the opposite surfaces of the opal films. By using the approach proposed by S. Reculusa et al. [42], considering in a first approximation, the inverse opal system as a layer of index *n_eff_* and of thickness *t* deposited on a glass substrate, and considering the wavelength difference of two consecutive FP maxima, the thickness (*t*) can be determined through expression (3):

(3) $$m\lambda_{p}\lambda_{p+m}=2n_{eff}\left(\lambda_{p+m}-\lambda_{p}\right)t$$

From Equation (3) it appears that the plot of $m\lambda_{p}\lambda_{p+m}$ versus $2n_{eff}\left(\lambda_{p+m}-\lambda_{p}\right)$ is a linear regression with a slope equal to the thickness *t* of the sample.

Considering the effective refractive index, determined using Equation (2) and the different *m*, applying a linear fit, the thickness *t* of the inverse opal was estimated to be about 8 μm.

The functionalization of the inverse silica opal structure was obtained via epoxy chemistry, and then, an amino-modified DNA-aptamer sequence labelled with Cy3 fluorophore was covalently bounded.

It is important to recall that in the last years, colloidal crystals have been exploited for the realization of biosensors in a dye-labeled fluorescence detection scheme [43]. However, the development of a robust, facile, stable, and cost-effective approach, which allows the uniform whole functionalization through the entire inverse structure, is still a challenge, considering its peculiar structural and morphological features. This step will permit the achievement of photonics-based biosensors, combining the optical (high density of states) and structural properties (high surface to volume ratio) of the colloidal crystals with the selectivity due to the specific probe immobilization.

Since the reflectance and transmission measurements on PCs do not provide full details of the dispersion relation ω_n_(k) directly, but rather they measure the number of the states’ available for a given direction of propagation, the numerical calculation of the DOS is a crucial step to discuss the rate of SE from the quantum emitters. In this context, although the silica opal structures have a weak effect on the total DOS as also reported in Reference [44], they produce a well-defined stop band in the direction of the light propagation (see Figure 2) and induce modification on the spontaneous emission of the fluorescent species embedded in the PC [45,46].

Thus, it is important to recall that the rate of spontaneous emission from an emitter, which undergoes a transition from initial state *i* to a final state *f*, can be expressed using Fermi’s golden rule:

(4) $$W\left(\omega^{\prime }\right)=\frac{2\pi }{h}{\left|\langle f|{\hat{H}}_{int}^{\prime }|i\rangle \right|}^{2}\cdot g\left(\omega^{\prime }\right)$$

where *h* is the Planck constant, ${\left|\langle f|{\hat{H}}_{int}^{\prime }|i\rangle \right|}^{2}$ is the squared modulus of the matrix element of the Hamiltonian of the radiation–substance interaction, and $g\left(\omega^{\prime }\right)$ corresponds to the density of optical states (DOS).

From Equation (4), it is possible to notice that the rate of SE is proportional to the density of states $g\left(\omega^{\prime }\right)$.

Now, a possible approach that allows the calculation of the photonic band structure and the resulting $g\left(\omega^{\prime }\right)$ for the opal structures is based on the H-field plane wave expansion (PWE) method [40]. The band diagram of the silica inverse opal structure, as calculated by the PWE method is reported in Figure 4a, where the crystalline symmetry points in the k space are reported in abscissa. The normal to the opal film surface was along the <111>-direction, equivalent to the L-point in the k-space. Starting from the band diagram, it was possible to calculate the DOS along a specific direction simply by counting the number of eigenstates at any specific frequency ω. The case of the Г-L k-space direction was expressed in the Equation (5) and using a delta-distribution:

(5) $$g\left(\omega \right)=\underset{n}{\sum }\underset{BZ_{\Gamma -L}}{\int }dk^{2}\cdot \left(\delta \left(\omega -\omega_{n}\left(k\right)\right)\right)$$

**Figure 5:**
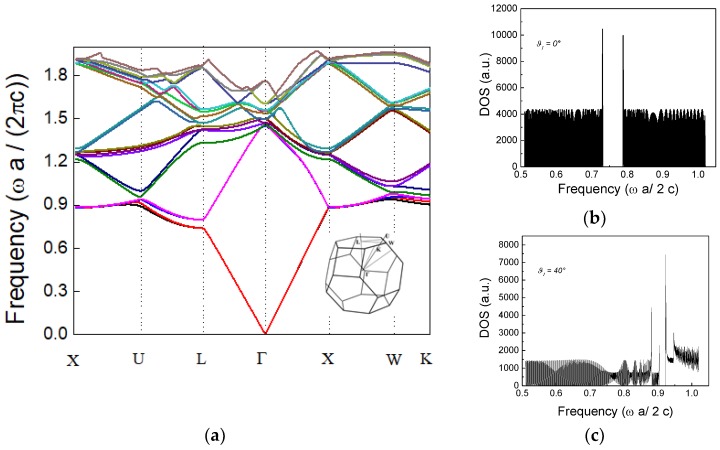
(**a**) Calculated band structure of the silica inverse opal. The inset is the reduced Brillouin zone. (**b**,**c**) Calculated DOS of inverse silica opal constituted by hollow spheres of about 370 nm at ϑ_i_ = 0° and ϑ_i_ = 40° to the direction Г-L, respectively.

Results for the DOS calculated in different directions at (a) normal incidence and (b) at 40° are shown in Figure 5b,c.

To verify the effective immobilization of DNA-aptamer-Cy3 on the network of the inverse silica opal, a confocal microscope was used. The fluorescence intensity at 575 nm (maximum Cy3 emission) of the ISO-aptamer-Cy3 was compared to the signal coming from a flat silica substrate functionalized in the same way, where an increase in the emission attributed to the immobilization of the aptamer-Cy3 sequence of about 25 times on the ISO structure was observed. Moreover, considering the intensity of the fluorescence signal in correspondence to its Full Width Half Maximum, it was possible to estimate that the signal came from a volume of about 10 μm (see Figure S1 in the ESI file), in agreement with the thickness value obtained by the optical measurements and the SEM images.

**Figure 6:**
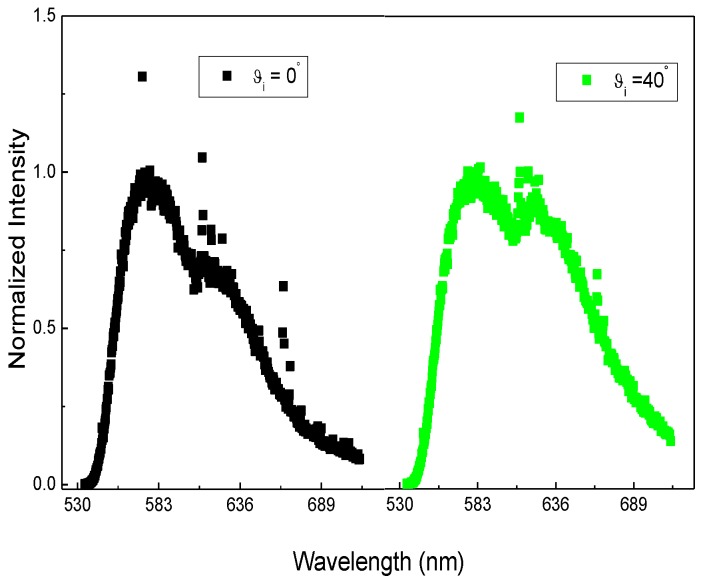
DNA-Cy3 fluorescence of the inverse opal obtained using an Ar^+^ line (514.5 nm) and collecting the emission at 0° and 40° detection angles.

Furthermore, starting from the above considerations, the immobilization of the DNA-aptamer-Cy3 on the silica matrix was also investigated considering the variation of the fluorescence signal as a function of the modification of the DOS as shown in Figure 6.

In fact, due to its periodic nature, the photonic crystal affected the fluorescence of the internal emitters (fluorophore) of the structure. As shown in Figure 4, the inverse opal had a pseudo-photonic bandgap (PBG) along the <111>-direction family. An immediate consequence was that the photons with energy that lay within the PBG could not propagate along the <111>-direction, and therefore, the spontaneous emission properties could be modified as described by Equation 4.

Tuning the position of the band gap of the inverse opal, which depended on the angle θ of incidence with respect to the <111> face, it was possible to overlap the PBG with the emission of the DNA-Cy3. Figure 6 shows the DNA-Cy3 fluorescence spectra obtained at different angles (a) 0° and (b) 40°, respectively.

Analyzing the spectra reported in Figure 6, it was possible to observe two main situations: (a) At 0°, there was no overlapping between the DNA-Cy3 and the PBG, and (b) At 40°, due to its blue shift, the photonic band gap (see Figure 3b) overlapped with the emission of the DNA-Cy3. A suppression of fluorescence was evident, strengthening the indication that the aptamer sequence was immobilized over the entire inverse structure.

Finally, to quantify the effect of the photonic crystal on the emission spectrum of the DNA-Cy3, the intensity ratio at 575 nm and 620 nm (I_620_/I_575_), for the spectra reported in Figure 6, was compared and an enhancement of 25% was determined. From this perspective, to observe a variation in the fluorescence emission intensity after target binding, the possibility to enhance the emission by changing the photonic environment could decrease the limit of detection of the whole system.

Moreover, it was worth mentioning that one of the main drawbacks of fluorescence-based sensors was the photo-bleaching of the organic dyes [47]. In this context, the exploitation of the features of the colloidal crystals and in particular the ability to modify the density of states could be used as a suitable solution. In fact, as evidenced in Figure 5b,c, at the band edge, choosing appropriately the exciting pumping angle, the photons propagated slowly through the photonic crystal. This allowed enhancement of the interaction with DNA-Cy3, thereby reducing the pump power and consequently the photobleaching effect. At the same time, the ability to immobilize a specific DNA-aptamer, highly reproducible in target recognition, on the surface of the inverse opal, paved the way for the realization of bio-sensors in the dye labelled fluorescence detection scheme.

Preliminary measurements, on the recognition of TNF, have been carried out by exploiting the fluorescence variation (Figures S3 and S4 in the ESI File), suggesting that the prepared DNA-aptamer inverse photonic crystal could be a suitable platform for the realization of biosensors in the dye labelled fluorescence detection scheme.

## 4. Conclusions

In this paper, we developed a versatile two steps approach for the realization of silica inverse opals functionalized with a DNA-aptamer sequence labelled with Cy3 fluorophore. The use of inverse silica opal due to its high porosity and its increased surface area permitted the immobilization of a higher number of emitters, with respect to the conventional planar surface. Through fluorescence measurements it was verified that the epoxy-chemistry approach effectively allowed the immobilization of the DNA-Cy3 sequence on the entire thickness of the inverse network. Moreover, by exploiting the modification of the DOS of the photonic crystal, a fluorescence enhancement of 25% was observed. The presented approach suggests that this kind of system can act as a suitable platform for the realization of biosensors in a dye labelled fluorescence detection scheme.

## Supplementary Materials

The following are available online at http://www.mdpi.com/1424-8220/18/12/4326/s1, Figure S1: Confocal analysis on aptamer-functionalized opal structure; Figure S2: SEM image of cross-section of opal structure; Figure S3: aptamer functionality; Figure S4: fluorescence detection of TNF-alpha molecule.
