# Supplementary material for: Fluorescent Aptamer Immobilization on Inverse Colloidal Crystals
Source: Sensors (Basel). 2018 Dec 7;18(12):4326. doi: 10.3390/s18124326 (PMC6308693; doi:10.3390/s18124326)
Supplement: Supplementary file 1 [file sensors-18-04326-s001.pdf]

Supplementary Information

Fluorescent Aptamer Immobilization on Inverse Colloidal Crystals

Andrea Chiappini [1], Laura Pasquardini [2,3,*], Somayeh Nodehi [4], Cristina Armellini [1], Nicola Bazzanella [5], Lorenzo Lunelli [2,6], Stefano Pelli [7,8], Maurizio Ferrari [1,8] and Silvia M. Pietralunga [4]

1 CNR-IFN CSMFO Lab. and FBK CMM, via alla Cascata 56/C, 38123 Povo Trento, Italy; andrea.chiappini@unitn.it (A.C.); cristina.armellini@unitn.it (C.A.); maurizio.ferrari@unitn.it (M.F.)
2 FBK-LaBSSAH, via Sommarive 18, 38123 Povo Trento, Italy; lunelli@fbk.eu
3 Indivenire Srl, via Alla Cascata 56/C, 38123 Povo Trento, Italy
4 CNR-IFN Milano, piazza Leonardo da Vinci 32, 20133 Milano, Italy; somayehnodehi@gmail.com (S.N.); silvia.pietralunga@polimi.it (S.M.P.)
5 Physics Department, University of Trento, via Sommarive 14, 38123 Trento, Italy; nicola.bazzanella@unitn.it
6 Institute of Biophysics, CNR, via Sommarive 18, 38123 Povo Trento, Italy
7 IFAC—CNR, MiPLab., via Madonna del Piano10, 50019 Sesto Fiorentino, Italy; s.pelli@ifac.cnr.it (S.P.)
8 Enrico Fermi Centre, piazza del Viminale 1, 00184 Roma, Italy
* Correspondence: l.pasquardini@gmail.com

1. The effective immobilization of DNA-aptamer-Cy3 on the network of the inverse silica opal is confirmed by confocal microscopy. Figure S1 reports the z-stacking profile and also the fluorescence distribution that provides an estimation of the opal thickness of about 10 µm.

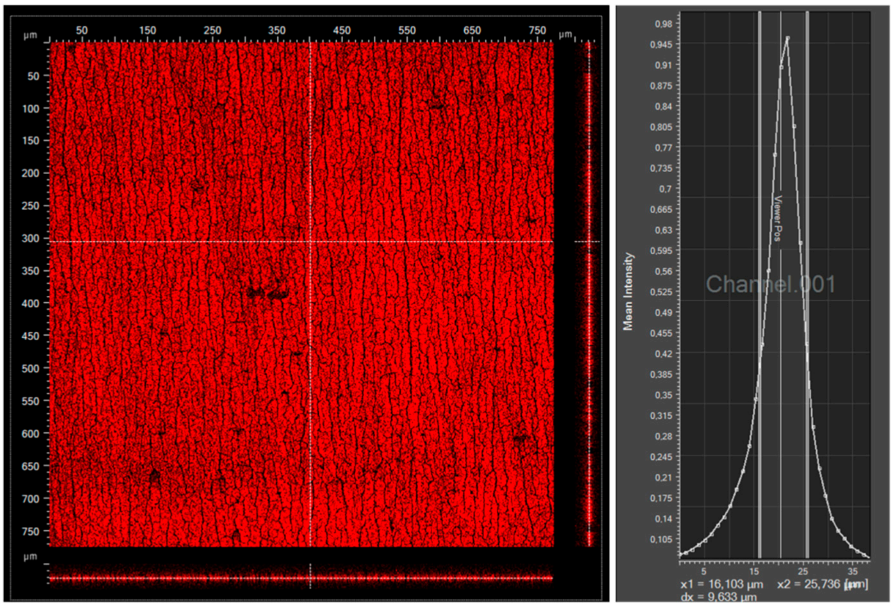

Figure S1. Cy3 fluorescence on aptamer-functionalized opal structure acquired with a z-stacking. The acquisition is performed using a 20X magnification objective, an HeNe laser 543nm with a power of 10.5 mW/cm^2. The distribution of the fluorescence signal is reported on the right.

Figure S2 reports the cross section of the inverse silica opal, where a thickness of about 8 μm has been measured.

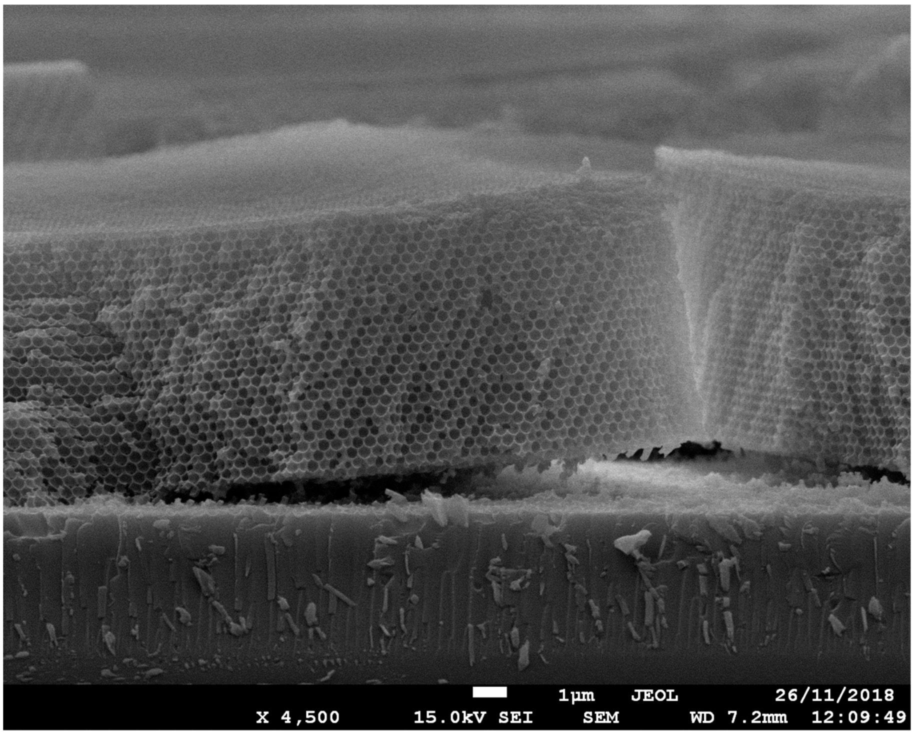

Figure S2. SEM image of the cross section of the inverse silica opal where a highly ordered structure is evident on 8 μm thickness.

2. As a proof of concept we tested our functionalized opal structure for the recognition of specific target. The utilized sequence (VR11) reported by Orava et al. [1] is specific for TNF-alpha recognition. We also utilized a second sequence in order to test the specificity of our system. The utilized control sequence is labeled "miD16" (5'-/5AmMC12/ TAGCAGCACGTAAATATTGGCG/3Cy3Sp/ -3'), with amino modification in the 5' end and Cy3 fluorophore in the 3' end. It is HPLC purified and is purchased from IDT Integrated DNA Technologies (Leuven, Belgium).

After the functionalization protocol reported in paragraph 2.4 of the main text, the prepared opal structures are incubated with the target protein. As reported by Kretschy et al. [2], Cy3 fluorophore is also quite sensitive to the particular nucleobase sequence of the ssDNA oligonucleotide to which they are attached, resulting in a quenching effect depending on the proximity of specific bases. In this perspective, once the aptamer recognizes his target, a rearrangement in the structure happens and this could affect the Cy3 fluorescence. To assess this issue, we incubated the two functionalized opal structures with 100 nM of TNF-alpha target molecule or 100nM of Bovine Serum Albumin, BSA, as negative control. Both proteins were diluted in 0.1M phosphate buffer + 0.15M NaCl pH 7.4. The fluorescence signal was acquired with a confocal microscope.

Figure S3 reports the results obtained incubating with the different solutions the opal structures functionalized with the two sequences. A first control is performed comparing the intensity of the fluorescence signal before and after incubation with a buffer solution. A small decrease of the fluorescence is observed on both structures, possibly due to a partial structuration of the aptameric sequence in buffer. The signal obtained after the buffer incubation is the starting fluorescence value to be used as reference. When the opal functionalized with the miD16 sequence is incubated with BSA or with TNF-alpha, a further decrease of the fluorescence signal is detected (Figure S3, left panel), but its statistically significance is not very high (p value near 0.3). On the

contrary, when the specific sequence VR11 is used (Figure S3, right panel), a statistical significant decrease is obtained when the incubation with TNF-alpha is performed (p-value < 0.01).

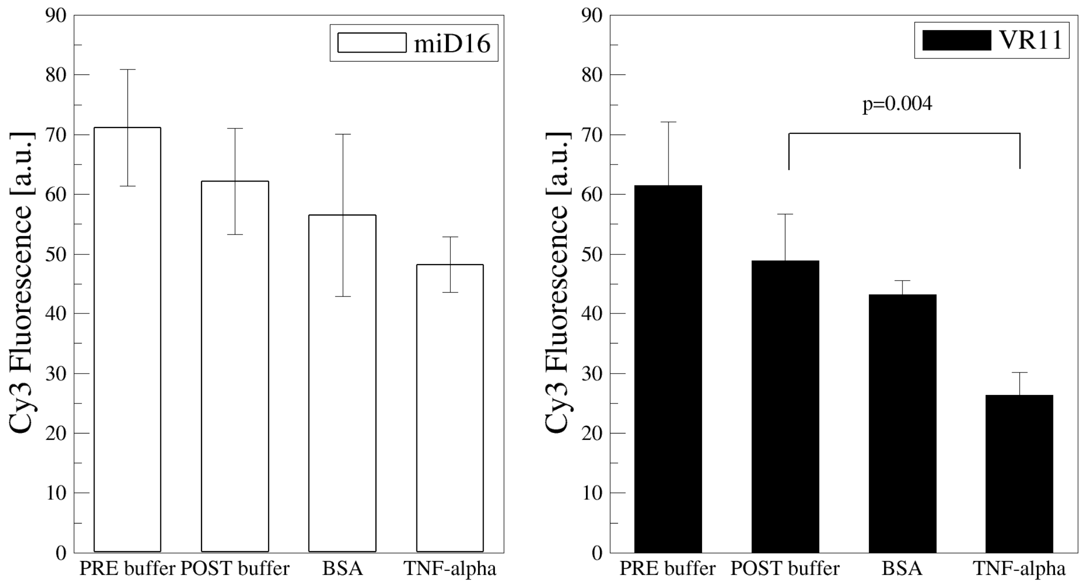

Figure S3. The Cy3 fluorescence signal was acquired before buffer incubation (PRE buffer), after incubation with a buffer solution (POST buffer), after the incubation with an aspecific (BSA) or specific (TNF-alpha) target protein. Two different aptameric sequences are immobilized on opal structures. On the left panel the result obtained using the miD16 sequence are shown, On the right panel, the VR11 sequence (specific for TNF-alpha recognition [1]) is used. The data are reported as mean value on four different opal structures for VR11 sequence and two for miD16. Error bars are reports as standard deviation. A t-test was applied to verify the statistical significance of the data).

3. To confirm the binging of the TNF-alpha on the VR11-functionalized opal structure, a NHS-Fluor (NHS-Fluorescein (5/6-carboxyfluorescein succinimidyl ester), mixed isomer, Thermo Fisher) is used to label free amine residues on the cytokine. NHS-Fluor at 50μM in phosphate buffer 0.1M pH 8 is applied for 3 hours, then washed in buffer and the fluorescence signal is acquired with a fluorescence microscope. The results are reported in Figure S4.

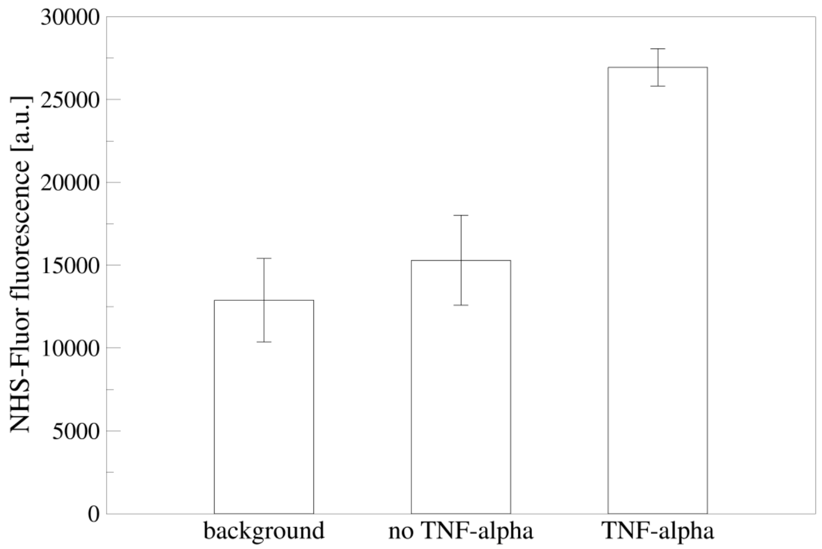

Figure S4. NHS-Fluor fluorescence on VR11-functionalized opal structure after incubation (or not) with TNF-alpha molecule. The value is reported as mean value on five images acquired on the sample and error bars represent the standard deviation. The "background" value reports the fluorescence signal only due to the opal structure.

References

1. Orava, E.W.; Jarvik, N.; Shek, Y.L.; Sidhu, S.S.; Garié, J. A Short DNA Aptamer That Recognizes TNFα and Blocks Its Activity. *ACS Chem. Biol.* **2013**, *8*, 170–178, doi:10.1021/cb3003557.
2. Kretschy, N.; Sack, M; Somoza, M.M. Sequence-Dependent Fluorescence of Cy3- and Cy5-Labeled Double-Stranded DNA. *Bioconjugate Chem.* **2016**, *27*, 840–848.
